# Regional Stress-Induced Ischemia in Non-fibrotic Hypertrophied Myocardium in Young HCM Patients

**DOI:** 10.1007/s00246-015-1214-5

**Published:** 2015-06-12

**Authors:** Robert Jablonowski, Eva Fernlund, Anthony H. Aletras, Henrik Engblom, Einar Heiberg, Petru Liuba, Håkan Arheden, Marcus Carlsson

**Affiliations:** Department of Clinical Sciences, Clinical Physiology, Lund University Hospital, Lund University, Lund, Sweden; Pediatric Heart Center, Lund University Hospital, Lund University, Lund, Sweden; Laboratory of Medical Informatics, School of Medicine, Aristotle University of Thessaloniki, Thessaloníki, Greece; Department of Biomedical Engineering, Faculty of Engineering, Lund University, Lund, Sweden; Centre for Mathematical Sciences, Faculty of Engineering, Lund University, Lund, Sweden

**Keywords:** Ischemia, Magnetic resonance imaging, Myocardial perfusion, Fibrosis, CMR

## Abstract

The relationship between hypertrophy, perfusion abnormalities and fibrosis is unknown in young patients with hypertrophic cardiomyopathy (HCM). Since mounting evidence suggests causal relationship between myocardial ischemia and major adverse cardiac events, we sought to investigate whether (1) regional myocardial perfusion is decreased in young HCM patients and in individuals at risk of HCM, and (2) hypoperfused areas are larger than areas with fibrosis. HCM patients (*n* = 12), HCM-risk subjects (*n* = 15) and controls (*n* = 9) were imaged on a 1.5 T MRI scanner. Myocardial hypertrophy was assessed on cine images. Perfusion images were acquired during adenosine hyperemia and at rest. Maximum upslope ratios of perfusion (stress/rest) were used for semiquantitative analysis. Fibrosis was assessed by late gadolinium enhancement (LGE). Results are presented as median and range. Perfusion in HCM-risk subjects and in non-hypertrophied segments in HCM patients showed no difference compared to controls (*P* = ns). Hypertrophic segments in HCM patients without LGE showed decreased perfusion compared to segments without hypertrophy [1.5 (1.1–2.3) vs. 2.0 (1.8–2.6), *P* < 0.001], and hypertrophic segments with LGE showed even lower perfusion using a segmental analysis [0.9 (0.6–1.8), *P* < 0.05]. The extent of hypoperfused myocardium in HCM patients during adenosine exceeded the extent of fibrosis on LGE [20 (0–48) vs. 4 (0–7) % slice area, *P* < 0.05] and hypoperfused areas at rest (*P* < 0.001). Regional perfusion is decreased in hypertrophied compared to non-hypertrophied myocardium and is lowest in fibrotic myocardium in young HCM patients but does not discriminate HCM-risk subjects from controls. The stress-induced hypoperfused regions exceed regions with LGE, indicating that hypoperfusion precedes fibrosis and may be a more sensitive marker of diseased myocardium in HCM.

## Introduction

Hypertrophic cardiomyopathy (HCM) is the most common inherited cardiac disease affecting one person in 500 and usually manifests in young adulthood [28]. Several genetic mutations, mainly of proteins coding of the cardiac sarcomere [26], have been linked to HCM. Patients with HCM can present with various symptoms ranging from dyspnea, palpations and fatigue to sudden cardiac death due to malignant arrhythmias [8, 9]. Therefore, identification of young patients with subclinical HCM and patients at risk of HCM is important. The morphological hallmarks of HCM are left ventricular (LV) diastolic failure due to hypertrophy and stiffening of the myocardium, decreased myocardial perfusion and fibrosis [2, 17, 35]. Cardiovascular magnetic resonance (CMR) has the ability to assess both LV thickness and volumes [25], presence of ischemia using stress CMR [35] and hyperenhancement on LGE-CMR [33], which has been shown to correspond to fibrosis in HCM [31]. The mechanisms and relation of the development of hypoperfused areas and fibrosis are, however, not fully understood. The genetic defect may cause a direct collagen deposition in the myocardium and vasculature causing hypoperfusion and myocardial fibrosis [16, 38]. An alternative hypothesis is that hypoperfusion occurs first with subsequent replacement fibrosis [4, 32]. The prognostic value of LGE in HCM remains elusive [11, 18], and ischemia has been proposed as an earlier marker of disease [4, 29, 32]. We have earlier showed that global perfusion is decreased in the same cohort of young patients with HCM investigated in the current study [12]. However, the relationship between regional perfusion, hypertrophy and fibrosis has not been studied in young HCM patients and HCM-risk subjects. An important question is whether these changes are present already at the very early stage of the disease, i.e., before the onset of myocardial hypertrophy.

Therefore, the aim of this study was to (1) measure if regional perfusion is decreased in young patients with HCM and in HCM-risk subjects and (2) determine whether hypoperfused areas are larger than areas with fibrosis.

## Materials and Methods

### Study Population

The study protocol conforms to the ethical guidelines of the Declaration of Helsinki and was approved by the Regional Ethical Review Board. Written informed consent was obtained from all subjects or, for subjects under 18 years of age, from their parents or legal guardians.

Patients <30 years of age were identified and recruited at the Departments of Pediatric and Adult Cardiology and referred for a CMR examination. Study participants were defined as either HCM patients or HCM-risk subjects upon inclusion using the following criteria: (1) HCM: if the patient had an interventricular septum (IVS) and/or posterior wall (PW) thickness exceeding 13 mm (subjects >18 years of age) or >3 SD on *Z*-score (pediatric patients) on echocardiography with confirmed increased wall thickness and/or fibrosis on CMR, (2) HCM-risk: if the subject had either a HCM gene mutation or first-degree relatives with HCM, but without signs of LV hypertrophy or fibrosis on CMR. Genetic testing was done according to guidelines by the clinic, and information of the HCM-causing mutation was collected from the medical record. Healthy age- and gender-matched controls (no history of cardiac disease, hypertension and normal 12-lead electrocardiogram) were also included.

Exclusion criteria were as follows: LV outflow tract obstruction, LV hypertrophy (LVH) due to other causes including congenital heart disease, malformation syndromes, neuromuscular and metabolic disorders. Subjects with contraindications for CMR were not enrolled. Typical contraindications for adenosine, which was used as a stress agent, such as allergic asthma and high-degree AV block, were followed. However, subjects not eligible for adenosine were included in the rest of the CMR protocol. All subjects were asked to refrain from caffeine 24 h prior to the CMR examination.

### Imaging Protocol

All CMR imaging was performed on a 1.5 T scanner (Philips Achieva, Best, The Netherlands) using a 32-channel coil. LV function was assessed by cine imaging using a steady-state free precession sequence (SSFP) in breath hold both in short-axis and long-axis projections (2-, 3- and 4-chamber views).

#### Regional Perfusion

Regional perfusion imaging was performed with a balanced turbo fast-field echo (TFE) sequence in breath hold (TR 2.7, TE 1.4, α 50°, acquired spatial resolution 2 × 2 × 10 mm reconstructed to 1.4 × 1.4 × 10 mm and SENSE factor 3). Images were acquired during the first pass of a bolus of a gadolinium (Gd)-based contrast agent (*Dotarem,* 0.05 mmol/kg, injection rate 5 ml/s) followed by a saline flush (injection rate 5 ml/s). Perfusion images were acquired in three short-axis slices at basal, midventricular and apical levels. Images were acquired during adenosine (140 μg/kg/min) hyperemia and at rest 10 min after terminating the adenosine infusion.

#### Late Gadolinium Enhancement

LGE-CMR images were acquired with a 3D inversion recovery gradient echo (IR GRE) sequence mid-diastole during end-expiratory breath hold. Short-axis slices covering the entire LV from base to apex and three long-axis projections were collected 10–20 min after administration of an additional 0.1 mmol/kg of *Dotarem* after the resting perfusion. Typical image parameters were as follows: five slices per breath hold reconstructed with no slice gap, slice thickness 8 mm and inplane resolution 1.5 × 1.5, echo time 1.3 ms, effective repetition time every heartbeat, flip angle 15° and inversion time 220–280 ms.

### Image Analysis

The software Segment v1.9 (http://segment.heiberg.se) was used for all image analysis [14].

#### Left Ventricular Dimensions

Left ventricular mass (LVM), global LV function and regional end-diastolic LV wall thickness were determined by first manually delineating the endocardium and epicardium in short-axis cine images at both end-systole and end-diastole. LVM was calculated by multiplying the myocardial volume measured by planimetry with the myocardial density (1.05 g/ml). The following LV indices were determined from the planimetric short-axis cine measurements: end-diastolic volume (EDV), end-systolic volume (ESV), stroke volume (SV) and ejection fraction (EF). The maximum end-diastolic wall thickness was measured in the IVS and PW in two short-axis slices.

#### Regional Perfusion

First-pass-perfusion images were delineated by semiautomatically tracing the endocardium and epicardium in all time frames during the first pass of the contrast bolus. The slices were divided according to the American Heart Association (AHA) 17-segment model with the apex being left out, yielding a maximum of 16 segments per subject with six basal, six midventricular and four apical segments. The delineated contours were contracted by 10 % both endo- and epicardially to account for partial volume and interaction with the blood pool. A semiquantitative measurement of maximum upslope ratio was used to assess the perfusion and was calculated as the ratio of perfusion stress and rest values normalized for the arterial input function in the LV blood pool [19]. In order to calculate maximal upslope, a linear curve fitting was applied automatically in a plugin to the software used.

Furthermore, co-registration between cine, LGE-CMR and perfusion short-axis images was performed in order to identify segments that were hypertrophied (LVH+) or of normal wall thickness (LVH−), hyperenhanced on LGE (LGE+) or not (LGE−). The upslope analysis was performed on a segmental basis and also on a per subject level by averaging all segments with the same tissue classification. An assessment of the hypoperfused area (expressed as an average percentage of the short-axis LV surface area for all perfusion slices) at rest and stress was performed and compared to the hyperenhanced region on the corresponding LGE-CMR short-axis slice.

#### Late Gadolinium Enhancement

Hyperenhancement on LGE-CMR was quantified on short-axis LGE images using a semiautomatic method with manual corrections where necessary [13].

### Statistical Analysis

Calculations and statistics were performed using Graph Pad Prism 5.0 software (Graph Pad Software, Inc., La Jolla, CA, USA). Results are expressed as median and range. The Kruskal–Wallis test with Dunn’s post hoc test was used to compare global LV parameters, regional wall thickness, fibrosis and myocardial perfusion between different groups and myocardial segments. Differences in upslope ratio corrected for LV arterial signal input was used to differentiate myocardial segments that were either LVH−LGE−, LVH+LGE− or LVH+LGE+. Differences with a *P* < 0.05 were considered statistically significant.

## Results

### Study Population Characteristics

Thirty-six young subjects were included: 12 patients with HCM [20 (12–30) years, two females], 15 subjects at risk of HCM [18 (14–26) years, seven females] and nine controls [21 (16–30) years, two females]. Patient characteristics are listed in Table 1. Genetic testing for known HCM mutations was available in eleven patients with HCM and revealed three patients with MYH7, three with MYBPC3 and one with TCAP, and in four subjects, no HCM mutation was found. In the HCM-risk group, eight subjects performed genetic testing (four MYBPC3, one TNNT2 and no HCM mutation was found in three subjects).

**Table 1:** Subject characteristics

	Controls (*n* = 9)	HCM-risk (*n* = 15)	HCM (*n* = 12)
Age (years)	21 (16–30)	18 (14–26)	20 (12–30)
Females, *n* (%)	2 (22 %)	7 (47 %)	2 (17 %)
β-blockers, *n*	0	0	4
End-diastolic volume/BSA (ml/m^2^)	101 (91–127)	92 (72–107)	91 (61–131)
End-systolic volume/BSA (ml/m^2^)	42 (38–63)	37 (29–49)	33 (23–84)*
Ejection fraction (%)	57 (42–63)	57 (52–66)	62 (36–77)
LVM/BSA (g/m^2^)	59 (37–74)	46 (37–56)	65 (43–158)^†^
Interventricular septum thickness (mm)	10 (6–11)	9 (7–11)	17 (14–36)*^,†^
Posterior wall thickness (mm)	8 (5–9)	7 (5–8)	8 (7–16)^†^

All values are expressed as median (range)

* *P* < 0.05 compared to controls; ^†^ *P* < 0.05 compared to HCM-risk

### Left Ventricular Hypertrophy

No areas of LVH were found in the control or HCM-risk group. LVH was found in all HCM patients, most frequently in the basal and/or midventricular anteroseptal (*n* = 11 patients) and inferoseptal wall (*n* = 9 patients).

### Regional Myocardial Perfusion and Fibrosis

In one HCM patient and one HCM-risk subject, no adenosine was administered due to the inability of securing an intravenous access in place and allergic asthma, respectively. Both were therefore excluded from the perfusion analysis. In three HCM patients and three HCM-risk subjects, only two short-axis perfusion slices were acquired due to high heart rate. Segments with a large mismatch between rest and stress images and segments with apparent artefacts were excluded (25 segments out of 510 in total).

**Fig. 1:**
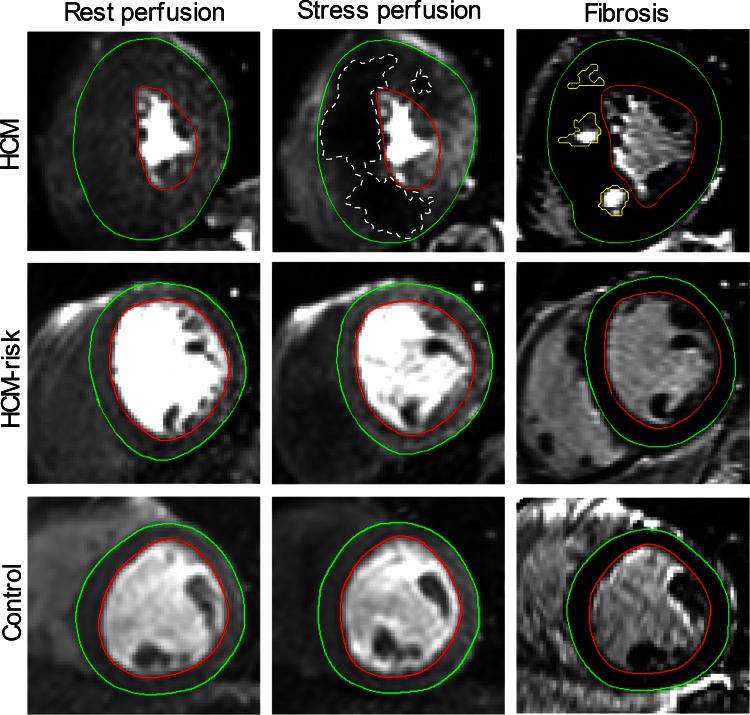
Short-axis first-pass perfusion images of the left ventricle at rest (*left column*), adenosine stress (*middle column*) and corresponding late gadolinium enhancement (LGE)-CMR image for determining fibrosis (*right column*) in one patient with hypertrophic cardiomyopathy (HCM), one HCM-risk subject and in one healthy control. In the HCM patient, the extent and severity of hypoperfused areas (*white dashed line*) were larger at adenosine stress compared to rest. The extent of hypoperfused areas at adenosine stress was also larger compared to fibrosis on LGE-CMR (*yellow line*), suggesting ischemia as the precursor of fibrosis. *Green line* = epicardium, *red line* = endocardium

Figure 1 shows representative perfusion images at rest and stress as well as corresponding LGE images in all three groups. No perfusion deficits were observed in HCM-risk subjects or in normal controls. In two HCM patients (18 %) a perfusion deficit was found at rest and in eight patients (73 %) during adenosine. One of these patients showed hypoperfusion during adenosine without any LGE in any segment with hypertrophy, i.e., only LVH+LGE− segments. The remaining patients with perfusion deficits (*n* = 7) had at least one LVH+LGE+ segment.

No hyperenhancement on LGE was present in HCM-risk subjects or controls. Eight HCM patients (73 %) showed hyperenhancement on LGE with a median of 2.9 (0.5–12) % scar of LVM. Of these, seven patients also had hypoperfused myocardium during adenosine. In one patient, the basal area with LVH+LGE+ was not obtained during perfusion imaging and no hypoperfused areas were visible in the LVH−LGE− slices.

#### Perfusion Reserve Index

**Fig. 2:**
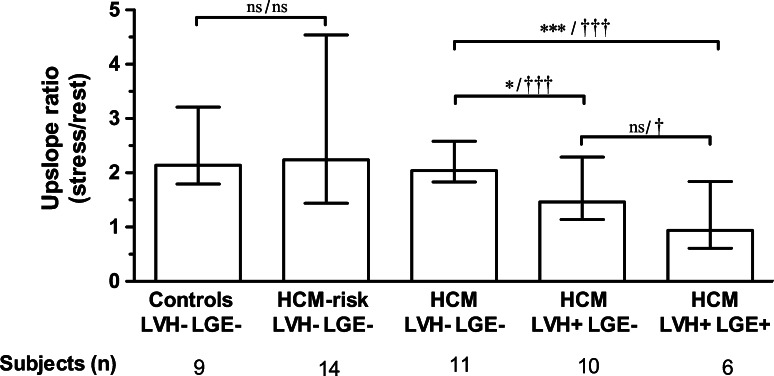
Regional myocardial perfusion expressed as upslope ratio (median, range) in normal controls, patients at risk of hypertrophic cardiomyopathy (HCM-risk) and HCM patients. All subjects were subdivided into three groups based on morphology: (1) LVH−LGE−: segments without left ventricular hypertrophy (LVH) and fibrosis (LGE), (2) LVH+LGE−: segments with LVH but without fibrosis and (3) LVH+LGE+: segments with LVH and fibrosis. There was a significant difference between the LVH−LGE− segments compared to both LVH+LGE− and LVH+LGE+ segments in HCM patients. Segments that were LVH+LGE+ had lower perfusion than LVH+LGE− segments and were statistically significant on segmental analysis but not averaged on a per-patient basis. No difference in regional perfusion was seen between HCM-risk subjects and controls. *P < 0.05, ***P < 0.001 per-patient analysis; ^†^ *P* < 0.05, ^†††^ *P* < 0.001 on segmental analysis

Semiquantitative analysis of perfusion expressed as the ratio between upslope of stress and rest perfusion is shown in Fig. 2. Analysis on a segmental level in HCM patients showed that LVH+LGE− segments had decreased perfusion (median, range) compared to LVH−LGE− segments [1.5 (1.1–2.3) vs. 2.0 (1.8–2.6), *P* < 0.001, *n* = 26 and *n* = 96 segments, respectively]. Segments with LVH+LGE+ showed even lower perfusion [0.9 (0.6–1.8), *n* = 27 segments] compared to LVH+LGE− segments (*P* < 0.05). When performing the analysis on a per-patient basis with segments averaged all differences remained statistically significant except the comparison between LVH+LGE− (*n* = 10 patients) and LVH+LGE+ (*n* = 6 patients, *P* = ns). There was no significant difference between HCM-risk subjects and LVH−LGE− segments in HCM patients compared to controls [2.2 (1.4–4.5), 2.0 (1.8–2.6) vs. 2.1 (1.8–3.2), *P* = ns]. However, both LVH+LGE− and LVH+LGE+ segments in HCM patients had lower perfusion compared to controls (*P* < 0.001).

#### Hypoperfused Areas Compared to Hyperenhancement on LGE-CMR

**Fig. 3:**
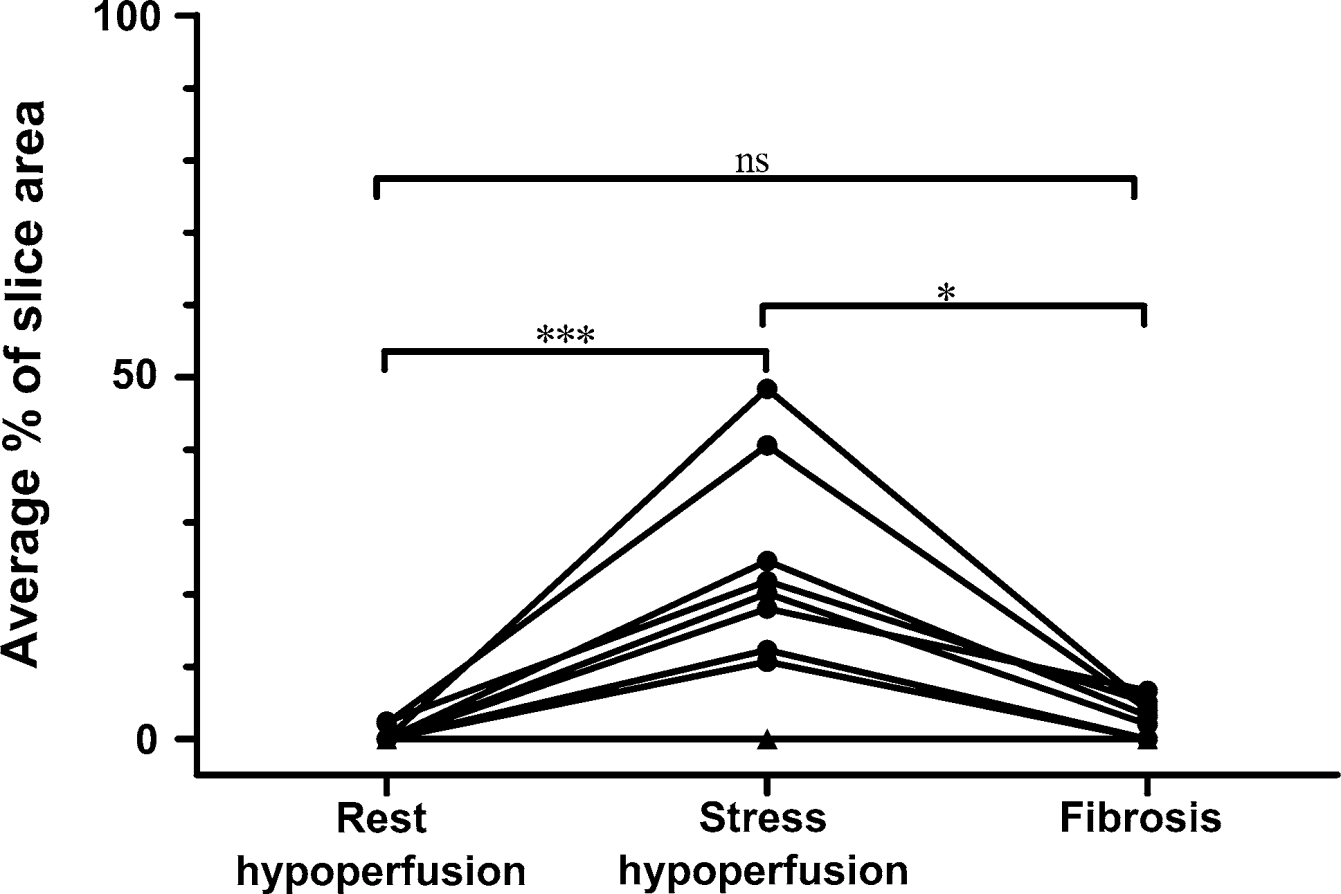
Areas of hypoperfusion in HCM patients at rest, adenosine stress and fibrosis determined as hyperenhancement on LGE-CMR expressed as an average % of slice area in all patients with HCM. Areas of hypoperfusion at adenosine stress are significantly larger than hypoperfused areas at rest and fibrotic areas. *Black triangles* = three patients overlapping with no perfusion deficits or fibrosis on LGE. *P < 0.05, ***P < 0.001

**Fig. 4:**
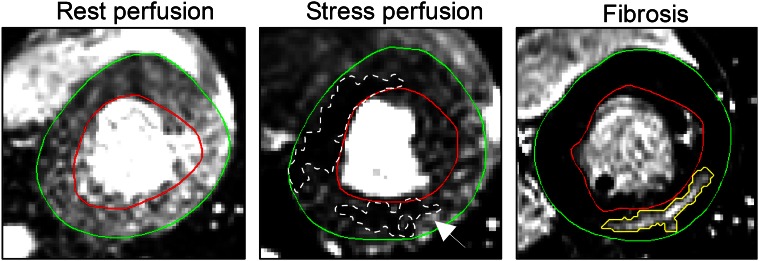
Short-axis first-pass perfusion images of a HCM patient at rest (*left column*), adenosine stress (*middle column*) and corresponding late gadolinium enhancement (LGE)-CMR image for determining fibrosis (*right column*), demonstrating two areas with hypoperfusion at stress perfusion. Interestingly, the area within the lateral wall (*white arrow*) shows a larger area of fibrosis (*yellow line*) than apparent hypoperfusion (*dashed white line*). This may be a display of fibrosis pathology of a different etiology. *Green line* = epicardium, *red line* = endocardium

The area of hypoperfused myocardium in HCM patients during adenosine [20 (0–48) % slice area] was larger compared to rest [0 (0–2) % slice area, *P* < 0.001; Fig. 3.] and was in all patients larger compared to the area of hyperenhancement on LGE [4 (0–7) % slice area, *P* < 0.05]. There was no significant difference between hypoperfused areas at rest and areas with LGE (*P* = ns). In all but one patient, areas with LGE were located in hypertrophied myocardium and within hypoperfused areas. The remaining patient had both areas with hypoperfusion exceeding areas with hyperenhancement on LGE, and LGE in the lateral wall with a borderline hypertrophied wall (12–13 mm) without a perfusion defect (Fig. 4).

## Discussion

This study indicates that young patients with HCM have regional stress-induced ischemia in hypertrophied myocardium. Perfusion during adenosine was even further decreased in myocardial segments with both hypertrophy and fibrosis compared to non-fibrotic hypertrophied segments. The extent of stress-induced hypoperfusion was larger compared to fibrosis, suggesting that ischemia may play a primary role in the pathogenesis of fibrosis in HCM. Young subjects at risk of HCM showed no difference in myocardial perfusion compared with controls.

### Comparison to Earlier Work

In the current study, we found lower perfusion in segments with LVH+LGE− and even lower perfusion in LVH+LGE+ segments, which, to our knowledge, has previously not been reported in young HCM patients. In cohorts with older HCM patients, similar degree of hypoperfusion in areas with LVH was found regardless of the presence of fibrosis [17, 21, 32, 34, 37]. We have previously shown global hypoperfusion using flow measurements in the coronary sinus in young HCM patients [12]. The present study extends these results, showing that regional hypoperfusion is linked to both LVH and fibrosis. The perfusion deficits ranged from subendocardial to transmural, but fibrosis was predominantly found in the mid-mural and in hypertrophied segments, similar to previous studies [7, 30]. Our results with the perfusion deficits mainly manifesting at adenosine stress show the importance and added value of performing myocardial perfusion at both rest and stress [21, 32, 34, 37].

We did not find a difference in perfusion between HCM-risk subjects and normal controls, which supports our previous findings on global perfusion [12]. Genotype-positive subjects with no sign of HCM morphology at age 18 have been shown to have a more benign clinical course [10]. Demonstrating a normal perfusion during adenosine stress may provide a more sensitive and accurate way to correctly classify these subjects as true phenotype negative as compared to only wall thickness. This remains to be proven by further studies.

### Pathophysiology of Fibrosis in HCM

The cause and timeline of fibrosis development in HCM is still somewhat unclear. LGE on CMR results from an expansion of extracellular volume in the myocardium [3] and may be caused by edema, necrosis or fibrosis, which all can occur in HCM patients [1, 27]. LGE on CMR has been shown to correlate with fibrosis on histopathology in HCM [23, 31]. In the current study, the hypoperfused area was larger and more severe at adenosine stress compared to both rest perfusion and to the extent of fibrosis in corresponding slices. Our findings suggest that replacement fibrosis is driven by ischemia in the hypertrophied LV, with the assumption that LGE represents fibrosis. This finding that ischemia precedes the development of replacement fibrosis is also supported by other studies using CMR [17, 21, 29, 32, 34, 37] and positron emission tomography (PET) [5, 36]. Histologic features of HCM such as myocyte disarray, intramural arteriolar dysplasia [4, 24, 27], interstitial fibrosis [23, 31] and a lower capillary density [20] within the hypertrophied myocardial segments demonstrate the pathophysiological substrates for ischemia.

Others, however, have shown data supporting that fibrosis is primary in HCM. In a histopathological study, Varnava et al. [38] found poor correlation between myocyte disarray and small vessel disease with the presence of fibrosis. Furthermore, there is preclinical evidence suggesting that pathways involved in fibrosis and collagen deposition are activated in HCM before the onset of pathological evidence of disease [22]. In a clinical study, Ho et al. [15] confirmed these preclinical findings when showing that biomarkers of an increased collagen I synthesis in the myocardium is present in genotype-positive HCM-risk individuals with normal wall thickness without LGE. Thus, the authors argued that fibrosis in HCM may be a direct consequence of sarcomere mutations and not due to ischemia. However, in this study, perfusion imaging was not performed. Bravo et al. [5] and Soler et al. [35] have shown, in subsets of their populations, presence of LGE in regions with normal perfusion, with [5] and even without LVH in HCM patients [35]. In our study, we also found one patient with fibrosis and no evidence of ischemia in a lateral wall with borderline hypertrophy (Fig. 4). In the hypertrophied inferior and inferoseptal wall of the same patient, a clear area of hypoperfusion at stress was visible with fibrosis in the same area within the hypoperfused area. This split image could represent two different pathophysiological explanations for fibrosis in HCM [5].

### Clinical Implications and Further Directions

This study in young patients with HCM showed the benefit of performing a CMR scan to assess function, fibrosis and perfusion with adenosine stress, in the same session. However, in the current European Society of Cardiology guidelines [9], CMR with LGE for assessment of anatomy, function and fibrosis only has a class IIa recommendation (evidence level B), whereas transthoracic echocardiography for measurement of diastolic wall thickness has a class I recommendation (evidence level C). Furthermore, presence of fibrosis on CMR has modest prognostic implications [11, 18] and data on the prognostic value of areas with increased extracellular volume (ECV) on T1-mapping are lacking. There is currently no mention of perfusion imaging on CMR in HCM in guidelines [9], but HCM patients with ischemia have been shown to have a worse clinical outcome [6].

In the current study, regional perfusion imaging demonstrates pathology beyond LVH and LGE. Thus, ischemia on CMR as demonstrated in the current study may have the potential to be an earlier biomarker of disease in HCM [17, 29, 32]. This will be elucidated further in the current cohort in an upcoming 3-year follow-up study. However, there is a need for larger clinical prospective studies to evaluate whether adenosine perfusion is a sufficiently strong predictor of disease severity and progression to alter clinical management.

### Limitations

The study population is small, and the study was therefore not powered sufficiently to detect small differences in perfusion indices between HCM-risk and controls. However, the HCM patient population was sufficient to detect differences in perfusion between different regions in the myocardium. All patients and subjects at risk did not want to undergo genetic testing, thus making comparison between genotypes and CMR indices difficult.

## Conclusions

In summary, this study conducted in young asymptomatic HCM patients has demonstrated a clear relationship between regional ischemia and myocardial hypertrophy, with further decrease in myocardial perfusion in hypertrophied segments with signs of fibrosis. Regional myocardial perfusion did, however, not discriminate between HCM-risk subjects and controls. Furthermore, the area of stress-induced ischemia exceeded the fibrosis, suggesting that development of replacement fibrosis is being preceded by ischemia. Adenosine perfusion imaging may aid in the risk stratification of young HCM patients; however, there is a need for larger prospective studies to show the clinical impact.
